# Supplementary material for: Automatic unsupervised respiratory analysis of infant respiratory inductance plethysmography signals
Source: PLoS One. 2020 Sep 11;15(9):e0238402. doi: 10.1371/journal.pone.0238402 (PMC7485851; doi:10.1371/journal.pone.0238402)
Supplement: Attachment — Submitted filename: Response to the Reviewers.docx [file pone.0238402.s001.docx]

Response to Reviewer #1

 The use of the term **‘gold standard’**. Thank you for this comment. We have replaced the term gold standard with ground truth data. We have eliminated the use of quotation marks when referring to ground truth data.

The use of the term **two-sided window**: We have replaced the term two-sided window with the term analysis window. We refer to the analysis window width instead of length. On page 9 we have added a description of the analysis window.

“Metrics were computed using rectangular analysis windows centered around a time n. The analysis windows advance by one sample at a time and provide estimates of the metrics for each sample. The width of the analysis window varied for each of the detectors as reported in Table 1. [reference 19, 25, and new reference 24]. ”

We have added a new Table 1 to describe the analysis windows used in each of the stages of signal processing. We have added reference [24] as a citation for new Table 1.

**Table 1. Analysis Windows**. Analysis window widths used in each stage of signal processing for the Automated Unsupervised Respiratory Analysis (AUREA). The table gives the values used for each analysis window width and provides a rationale. Legend ASB = asynchrony, PAU = pause, MVT = movement, RIP = respiratory inductance plethysmography, SYB = synchronous.

New Table 1

| Window | Analysis Window Width  (s) | **Description of and Rationale for Analysis Window Width** |
| --- | --- | --- |
| **Preprocessing of RIP signals** | | |
| $N_{LF}$ | 5 | Analysis window width used for initial high-pass filtering of RIP signals. Based on previous work in ref 19 and 25. |
| **PAU Metric** | | |
| $N_{QV}$ | 120 | Analysis window width for computing the quantiles. We observed that infants have a SYB pattern more than 60 % of the time. Thus, we considered that a normalization quantile equal to the median should provide an appropriate reference to normal SYB values and a normalization analysis window of 2 minutes. |
| $N_{V}$ | 1 | Analysis window width for the estimate of variance; chosen empirically to provide for the fast response needed to detect short respiratory pauses. |
| $N_{DT}$ | 5 | Analysis window width for the removal of low frequency components. Based on previous work in ref 19 and 25. |
| **MVT Metrics** | | |
| $N_{MA}$ | 1.42 | Analysis window width used for the moving average notch filter. This width was selected such that the filter nulls were at harmonics of the most frequent respiratory frequency, which we defined as the mode of the respiratory frequency histogram, computed from the entire data set. This frequency was 0.7 Hz or 42 breaths per minute. |
| $N_{QRMS}$ | 600 | Analysis window width for computing the quantiles variance in the RIP signals. Based on results in ref 19 and 25. |
| $N_{RMS}$ | 5 | Analysis window width for computing RMS values of RIP signals. Based on results in ref 19 and 25. |
| $N_{DT}$ | 5 | Based on results in ref 19 and 25. |
| **SYB and ASB Metrics** | | |
| $N_{SMO}$ | 0.42 | Analysis window width for the smoothing window used in computing SYB and ASB metrics. Based on results in ref 24. |
| $N_{B}$ | 2 | Analysis window width used to compute the power of the SUM and DIFF signals. Based on results in ref 24. |
| $N_{DT}$ | 2 | Based on results in ref 24. |

[19] A. A. Aoude, R. E. Kearney, K. A. Brown, H. L. Galiana, and C. A. Robles-Rubio,

"Automated Off-Line Respiratory Event Detection for the Study of Postoperative Apnea in

Infants," *IEEE Trans Biomed Eng,* vol. 58, pp. 1724-1733, Jun 2011.

NEW [24]C. A. Robles-Rubio, K. A. Brown, and R. E. Kearney, "Detection of Breathing Segmentsin Respiratory Signals," in *Conf. Proc. 34th IEEE Eng. Med. Biol. Soc.*, San Diego, USA,2012, pp. 6333-6336.

[25] C. A. Robles-Rubio, K. A. Brown, and R. E. Kearney, "A New Movement Artifact

Detector for Photoplethysmographic Signals," in *Conf. Proc. 35th IEEE Eng. Med. Biol.*

*Soc.*, Osaka, Japan, 2013, pp. 2295 - 2299.

**Page 7** “These reference segments, totaled 2000s per second” Thank you for this constructive comment. We have reworded this section to read:

“Signals were recorded continuously at a sampling rate of 50Hz/channel, over 5 to 12 hours of observation during which the infants exhibited a range of behavior including feeding, crying, and sleeping. The total length of the reference signals inserted into each record was 2,000s (about 10% of the total record length) and..”

Page 8-9 **The decision tree**. We agree that description of the detectors as a decision tree adds clarity, and have borrowed some of your wording. The section has been revised as follows:

“In the second stage, AUREA uses a series of binary K-means classifiers to assign each sample to a pattern that is determined by combining the output of the pattern detectors as a decision tree. The decision tree, engages the pattern detectors, sequentially, to detect the class/anti-class pairs. If the decision is set to the target class, the tree has reached a leaf and the classification is over. If the decision is set to the anti-class, the sample moves to the next tree node where another binary classification is performed. The order of the class/anti-class pairs in this decision tree is PAU, MVT, SYB, ASB. The PAU detector has the highest precedence and is engaged first; the other detectors are disengaged. The rationale for the high precedence for PAU detector was that during pauses the power in the RIP signals is very low and as a result the variance of the MVT, SYB, and ASB metrics would be high leading to many false positives. The MVT detector was engaged next since the low frequency power associated with movement would bias the SYB and ASB detectors.”

Note that in the development of AUREA we did try reordering the sequence of binary classifiers. In ref [19] the order was PAU­-MVT- ASB-SYB. We also tried MVT-PAU-SYB-ASB, but this order performed worse than PAU-MVT-SYB-ASB. These results are not presented.

Page 11 **Preprocessing of signals**. We have replaced the term low frequency trends with low frequency components.

“The RIP signals were preprocessed to remove the mean value and low frequency components with a segment level mean normalization using an analysis window of width N_LF_.” In addition we have changed the word trend and subscript _TR_ for the words low frequency and the subscript _LF_ in Equation 1.

Page 12 **detrended RIP signals** We have removed the word detrended and refer to preprocessed RIP signals.

Page 12 **sample-by-sample variance**. Thank you for this comment. We have rephrased this section as follows:

“. As the analysis window advanced one sample at a time, there was an estimate for the variance at every analysis window. The normalized variance metric of the RIP signal was computed from the estimated variance at each sample using an analysis window of width as...” *Equation 3 on page 10.”*

Page 13 **stochastic periodic noise**. We have corrected this to read: “stochastic nonperiodic noise”.

Page 13 **Analysis window length**: See above and new Table 1.

**Figures**: we use the convention Fig # to identify figures.

**References** We have unified the style for references to include the bracket before the closing period.

**Missing spaces**. I have a silly version of WORD that misses spaces. I revised the manuscript on a different computer and think I have corrected all the missing spaces. I have edited et al. to include the period.

**Figures**: We have revised the figures 4-7, 12 and 15 to use unique trend lines that are distinguishable for black and white copies, and for the color blind.
